# Self-care practices and associated factors among hypertensive patients at public hospitals in North Shewa zone, Ethiopia

**DOI:** 10.3389/fmed.2024.1482061

**Published:** 2024-10-30

**Authors:** Hailemelekot Bekele Kebede, Tewodros Yosef, Awraris Hailu Bilchut, Sewnet Getaye Workie, Nigusie Shifera, Alemnew Destaw Mezgebu

**Affiliations:** ^1^School of Public Health, Asrat Woldeyes Health Science Campus, Debre Berhan University, Debre Berhan, Ethiopia; ^2^School of Public Health, College of Medicine and Health Sciences, Mizan-Tepi University, Mizan-Teferi, Ethiopia; ^3^School of Medicine, Faculty of Health, Deakin University, Waurn Ponds, Geelong, VIC, Australia

**Keywords:** hypertension, self-care practices, public hospitals, North Shewa, Ethiopia

## Abstract

**Background:**

Hypertension significantly contributes to premature mortality worldwide, highlighting the need for effective self-care to manage its complications. However, there is limited research on self-care practices among hypertensive patients in Ethiopia. This study assessed self-care practices and associated factors in hypertensive patients at public hospitals in North Shewa zone, Amhara Region, Ethiopia.

**Methods:**

A hospital-based cross-sectional study was conducted with 450 participants using multi-stage sampling and interviewer-administered questionnaires. Data were processed with Epi-Data 4.6.0.6 and analyzed using SPSS 23. Descriptive statistics summarized the results, while bivariable and multivariable logistic regression identified factors associated with self-care practices. Crude and adjusted odds ratios with 95% confidence intervals were calculated, with significance at *p* < 0.05.

**Results:**

Out of 450 participants, 231 (51.3, 95% CI: 46.4–55.6%) exhibited poor hypertension self-care practices. Factors associated with poor self-care included having a college-level education (AOR = 0.27, 95% CI: 0.07–0.95), university-level education (AOR = 0.36, 95% CI: 0.13–0.98), being widowed/widower (AOR = 5.30, 95% CI: 1.05–27.2), poor knowledge of hypertension (AOR = 4.51, 95% CI: 2.44–8.59), inadequate stress management (AOR = 3.10, 95% CI: 1.64–5.74), and first diagnosis during a check-up (AOR = 7.72, 95% CI: 4.22–13.8).

**Conclusion:**

This study highlights inadequate self-care among hypertensive individuals, affected by factors such as education, marital status, knowledge, stress management, and diagnostic practices. Personalized interventions focusing on health education, stress management, and proactive screening are essential for improving health outcomes. Additionally, psychological support enhances emotional well-being and self-care engagement in hypertension patients, leading to better health outcomes and quality of life.

## Introduction

Hypertension, or elevated blood pressure, is a serious medical condition that raises the risk of heart, brain, kidney disease, and other diseases (1). It is diagnosed when systolic blood pressure (SBP) is ≥140 mm Hg and/or diastolic blood pressure (DBP) is ≥90 mm Hg after several measurements (2). Hypertension is the leading noncommunicable disease (NCD), affecting about 31.1% of adults globally (1). It is present in 28.5% of cases in developed countries and 31.5% in developing regions, with low-and middle-income countries (LMICs) like Ethiopia facing a significant NCD burden (3).

In sub-Saharan Africa, approximately 74.7 million people have hypertension, with the number expected to increase by 68% by 2025 (4). Hypertension affects 25.9% of various population groups in the region (5). In Ethiopia, the nationwide incidence of hypertension was 19.6% in 2015 (6), with regional variations: 18.8% in Sidama (7), 28.3% in Gondar (8), 13.2% in Jimma (9), and 11% in Mekelle (10). Self-care practices like regular exercise, a heart-healthy diet, reducing salt, maintaining weight, limiting alcohol, quitting smoking, and taking medication are key to reducing hypertension and managing blood pressure. Embracing these measures is essential for controlling hypertension and reducing related morbidity and mortality (11, 12).

Adhering to self-care practices lowers blood pressure, enhances the effectiveness of medications, and significantly decreases complications and mortality associated with hypertension (13, 14). Improving self-care behaviors can greatly reduce the life-threatening complications of hypertension (15). Factors associated with poor hypertension self-care include low education (16–20), insufficient knowledge of hypertension (18, 21–23), poor stress management (18), routine hypertension diagnosis (24), social isolation (18), economic constraints (25), and comorbidities (22).

Previous research has explored lifestyle modifications, but many studies have not included key components recommended by the Joint National Committee for hypertension prevention, detection, and screening. In Ethiopia, earlier studies have largely focused on individual healthcare facilities, potentially limiting a comprehensive assessment of hypertension self-care practices. Additionally, there is a notable lack of zonal-level studies that include both woreda and tertiary hospitals (26–28). The COVID-19 pandemic further disrupted health systems, particularly affecting follow-up care for chronic conditions like hypertension in developing countries, including Ethiopia (29–31). This study aimed to address these gaps by examining self-care practices and identifying factors associated with hypertension among patients at North Shewa zone public hospitals in Ethiopia.

## Methods

### Study design, setting, and period

This research employed a hospital-based cross-sectional study conducted in the North Shewa zone, one of the 13 zones in the Amhara region of Ethiopia. The North Shewa zone, with Debre Berhan as its administrative center, is located approximately 147 kilometers north of Addis Ababa and 284 kilometers from Bahir Dar, the capital of the Amhara Regional State. The zone comprises 24 administrative districts, which include 10 hospitals, 99 health centers, and 446 health posts. Data collection for this study took place in public hospitals across the North Shewa zone from June to August 2022.

### Participants

The source population comprised all adult hypertensive patients visiting public hospitals in the North Shewa zone. The study population consisted of randomly selected hypertensive patients. Inclusion criteria were being 18 years or older and having been under follow-up for at least 6 months. Individuals with severe illness or auditory and speech impairments were excluded.

### Study variables

The dependent variable was self-care practices among hypertensive patients. The independent variables included sociodemographic factors (gender, age, religion, marital status, residence, education, and occupation) and clinical and behavioral factors (body weight, blood pressure diagnosis context, distance to healthcare facilities, comorbidities, khat chewing, education on noncommunicable diseases), as well as knowledge about hypertension and stress management ability.

### Sample size determination and sampling technique

The sample size was calculated using a single population proportion formula with a 95% confidence interval, 5% margin of error, and a 24% self-care practice rate from a prior study (26).


$$n=\frac{{\left(Z\;\alpha /2\right)}^{2}\;p\left(1-p\right)}{d^{2}}=\frac{{\left(1.96\right)}^{2}\;0.24\left(1-0.24\right)}{{\left(0.05\right)}^{2}}=280$$

Adjustments were made for a 10% nonresponse rate and a design effect of 1.5, resulting in a final sample size of 462. A multi-stage sampling technique was employed in this study. First, 3 out of 10 public hospitals in North Shewa zone were randomly selected using a lottery method. Next, a list of hypertensive patients from these hospitals’ follow-up unit registration books was created. Patients were then systematically sampled, with every second patient selected until the target sample size was achieved. Participants who missed three consecutive screenings were classified as nonresponses.

### Data collection methods and quality assurance

Primary data were collected using interviewer-administered questionnaires adapted from standardized tools employed by previous researchers (18, 32–34). The questionnaire comprised four components: sociodemographic factors, health status questions, self-care practices evaluated through the Hypertension Self-Care Activity Level Effects (H-SCALE), hypertension knowledge measured using the Hypertension Knowledge-Level Scale (HK-LS), and stress management assessed with the Perceived Stress Scale (PSS). The questionnaire was translated into Amharic and then back-translated into English by experts to ensure accuracy. Blood pressure measurement scales were calibrated prior to each reading and the average of two consecutive measurements were recorded. The internal consistency of the tools was evaluated through reliability testing (Cronbach’s alpha), yielding satisfactory results: 89.5% for the H-SCALE, 84% for the PSS, and 82% for the HK-LS. To ensure data quality, a pretest was conducted on 5% of the sample not included in the study, and data collectors received thorough training. Data collection was conducted by 8 diploma nurses and supervised by 2 bachelor degree nurses who received 2 days of intensive training.

### Operational definitions

Hypertension was defined based on SBP readings of 140 mmHg or higher and/or DBP readings of 90 mmHg or higher (2).

*Good self-care practice*: A hypertensive patient was considered to have good self-care if they adhered to at least four out of the six H-SCALE subscales (18).

*Poor self-care practice*: A hypertensive patient was considered to have inadequate self-care if they adhered to three or fewer H-SCALE subscales (18).

*Knowledge of hypertension*: Participants’ knowledge of hypertension was assessed using 22 yes/no items of HK-LS. Each correct response was scored as 1, and incorrect as 0. Scores were totaled, with a mean score of 11. Participants scoring 11 or above were classified as having “good knowledge,” while those scoring below 11 were classified as having “poor knowledge” (33).

*Stress management*: The PSS has 14 items, with seven reverse scored to assess lack of control and negative emotional reactions, and seven directly measuring coping with stressors. Responses are rated on a five-point Likert scale from 0 to 4. Total scores are summed, with scores at or above the mean of 7 indicating “good stress management,” and scores below the mean indicating “poor stress management.”

### Statistical analysis

First, the data were manually reviewed to ensure completeness and consistency. The completed questionnaires were then coded, entered into Epi-Data version 4.6.0.6, and analyzed using SPSS version 23. Descriptive statistics, including frequency, percentage, mean, and standard deviation, were used to summarize the results. Bivariable logistic regression was employed to assess the associations between predictors and outcome variables, calculating crude odds ratios and 95% confidence intervals. Independent variables with *p*-values ≤0.2 in the bivariable analysis were included in the multivariable analysis. The goodness of fit of the model was evaluated using the Hosmer–Lemeshow test (*p* value ≥0.05), indicating a well-fitting model. Multicollinearity among independent variables was assessed using variance inflation factors (VIFs <4 for all), ensuring tolerable correlations. Adjusted odds ratios (AOR) with a 95% confidence interval were used to identify factors associated with self-care practices, with statistical significance set at *p* < 0.05.

## Results

### Sociodemographic characteristics

Out of 462 individuals, 450 responded to the questionnaire, resulting in a strong response rate of 97%. The majority of respondents were male, comprising 52.7% (237 individuals). The average age was 39.8 years (±SD 8.9), with 50.4% (227 participants) in the 35–44 age range. Over half of the participants lived in urban areas (54.7%, 246 individuals). Additionally, one-third of the participants had no formal education (35.3%) (Table 1).

**Table 1:** Sociodemographic characteristics of study participants.

Variables	Category	Frequency (*n*)	Percent (%)
Gender	Male	237	52.7
	Female	213	47.3
Age group (yr)	21–34	139	30.9
	35–44	227	50.4
	≥45	84	18.7
Religion	Orthodox	311	69.1
	Muslims	102	22.7
	Protestant	30	6.7
	Catholic	7	1.6
Marital status	Single	42	9.3
	Married	323	71.8
	Divorced	54	12.0
	Widowed/widower	31	6.9
Residence	Rural	142	31.6
	Semiurban	62	13.8
	Urban	246	54.7
Education	No formal education	159	35.3
	Primary education	69	15.3
	Secondary education	46	10.2
	College education	114	25.3
	University and above	62	13.9
Occupational status	Civil servant	115	25.6
	Private employee	33	7.3
	House-wife	64	14.2
	Retired	35	7.8
	Farmer	100	22.2
	Merchant	103	22.9

### Clinical and behavioral characteristics

Approximately half of the participants (51.6%, 232) were of normal weight, while 44.2% (199) were classified as overweight. Blood pressure was diagnosed during routine check-ups for 48.7% (219) of participants, with one-third of them (36.7%, 165) having to travel over an hour to reach the hospital. Comorbidities were present in 28.4% (128) of participants, and 13.1% (59) were khat chewers. Additionally, 43.3% (195) had received education on noncommunicable diseases at the hospital.

### Self-care practices using H-SCALE

**Figure 1:**
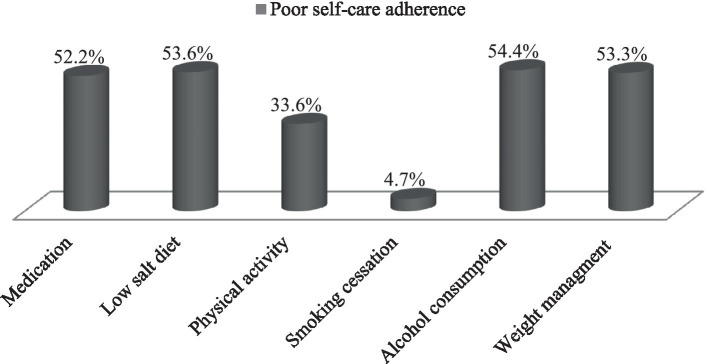
Study participants self-care adherence using H-SCALE.

Self-care activities among hypertensive patients were evaluated using the H-SCALE domains. Of the participants, 231 (51.3, 95% CI: 46.4–55.6%) demonstrated poor self-care practices related to hypertension. Of the 450 participants, over half (235, 52.2%) had poor medication adherence, and 241 (53.6%) had poor adherence to a low-salt diet. One-third (151, 33.6%) displayed poor adherence to physical activity guidelines. In contrast, only 21 (4.7%) had poor adherence to smoking cessation efforts. More than half of the participants (245, 54.4%) showed poor adherence to recommended alcohol consumption limits, and 240 (53.3%) exhibited poor adherence to weight management strategies (Figure 1).

### Factors associated with self-care practices

After adjusting for potential confounding variables, higher education, being widowed/widower, poor self-care knowledge, inadequate stress management, and first diagnosis of hypertension during a check-up were identified as significant factors associated with poor hypertension self-care practices. Participants with a college-level education were 73% less likely to exhibit poor self-care practices compared to those with no formal education (AOR = 0.27, 95% CI = 0.07–0.95). Those with university-level education or higher were 64% less likely to show poor self-care practices (AOR = 0.36, 95%CI: 0.13–0.98). Widowed/widower individuals were 5.3 times more likely to engage in poor self-care compared to single individuals (AOR = 5.30, 95%CI: 1.05–27.2). Poor knowledge increased the likelihood of poor self-care practices by 4.5 times (AOR = 4.51, 95%CI: 2.44–8.59), and poor stress management was associated with a threefold increase in poor self-care practices (AOR = 3.10, 95%CI:1.64–5.74). Additionally, participants first diagnosed with hypertension during routine check-ups were 7.7 times more likely to exhibit poor self-care practices compared to those who developed complications later (AOR = 7.72, 95% CI: 4.22–13.8) (Table 2).

**Table 2:** Factors associated with self-care practices among study participants.

Variables	Categories	Self-care practice		COR (95% CI)	AOR (95% CI)	*p*-value
		Poor	Good			
Age group (yr)	21–34	61	78	1	1	
	35–44	123	104	0.65 (0.43, 0.98)	0.62 (0.36, 1.23)	0.214
	>45	47	37	2.12 (0.97, 4.19)	0.94 (0.30, 3.14)	0.973
Marital status	Single	23	19	1	1	
	Married	167	156	1.16 (0.59, 2.15)	2.23 (0.77, 5.96)	0.146
	Divorced	31	23	0.83 (0.39, 2.02)	1.81 (0.52, 6.41)	0.335
	Widowed/widower	10	21	2.56 (0.96, 6.69)	5.30 (1.05, 27.2)	0.040
Education	No formal education	101	58	1	1	
	Primary education	42	27	1.14 (0.62, 2.00)	0.63 (0.22, 1.94)	0.457
	Secondary education	30	16	0.91 (0.46, 1.84)	0.77 (0.24, 2.53)	0.683
	College education	43	71	2.83 (1.74, 4.73)	0.27 (0.07, 0.95)	0.042
	University & above	15	47	5.42 (2.80, 10.6)	0.36 (0.13, 0.98)	0.041
Knowledge of hypertension	Poor	78	189	12.1 (7.74, 19.8)	4.51 (2.44, 8.59)	<0.001
	Good	153	30	1	1	
Stress management	Poor	58	173	11.1 (7.22, 17.4)	3.10 (1.64, 5.74)	<0.001
	Good	173	46	1	1	
Hypertension diagnosis time	During check-up	43	176	17.3 (11.1, 28.6)	7.72 (4.22, 13.8)	<0.001
	Post-complication	188	43	1	1	
Comorbidities	Yes	81	47	0.53 (0.33, 0.77)	0.65 (0.33, 1.27)	0.208
	No	150	172	1	1	
Khat chewing	Yes	37	22	0.58 (0.33, 1.02)	1.64 (0.75, 3.74)	0.212
	No	194	197	1	1	

CI, confidence interval; COR, crude odds ratio; AOR, adjusted odds ratio. * *p* < 0.05; ** *p* < 0.001.

## Discussion

In this study, a significant proportion of participants (51.3, 95% CI: 46.4–55.6%) exhibited poor self-care practices for managing hypertension. This finding is consistent with 48.5% in Addis Ababa (16), 51% in Dessie (17), 53.1% in Jimma (35) and 48% in Dire Dawa (21) studies in Ethiopia. These results highlight a troubling prevalence of inadequate self-care among hypertensive patients, identifying a critical area for intervention. Despite the established importance of self-care in managing hypertension and mitigating related complications, this issue remains significant (11, 12). Addressing this disparity is crucial for optimizing hypertension management and reducing the burden of non-communicable diseases related to hypertension.

Participants with a college-level education were 73% less likely to have poor self-care practices, while those with a university-level education or higher were 64% less likely to exhibit poor self-care compared to those without formal education. These findings are consistent with studies conducted in Ethiopia (16–18), Ghana (19), and India (20). Higher education enhances understanding of hypertension and self-care, improves health literacy, and boosts motivation for healthy behaviors. Educated individuals are better at managing their health, accessing resources, and often have higher socio-economic status, which provides better access to healthcare, healthy food, and physical activity, leading to improved hypertension management. This highlights the need for targeted educational interventions to improve awareness and self-care practices, helping to reduce disparities and enhance health outcomes across different educational levels.

Widowed or widower adults were 5.3 times more likely to have poor self-care practices for hypertension compared to single adults. This finding is consistent with a study in Dessie, Ethiopia (17). The stress and emotional strain of losing a spouse can hinder effective health management, reducing motivation and adherence to self-care. Widowed individuals may lack social support, experience loneliness, and face disrupted routines, all of which can negatively impact their ability to manage hypertension. Community programs and social support networks could help alleviate these effects and improve hypertension management outcomes for this group.

In this study, individuals with poor knowledge about hypertension were 4.5 times more likely to exhibit poor self-care practices compared to those with good knowledge. This finding is supported by studies conducted in Ethiopia (18, 21–23). Poor knowledge about hypertension can lead to neglect of self-care, misunderstanding of treatment guidelines, and difficulty recognizing symptoms. This lack of understanding reduces motivation and impairs decision-making, affecting overall management of the condition. Recommendations include tailored educational programs, detailed information on hypertension management, and community-based support groups to reinforce knowledge and adherence to self-care practices.

The study found a significant link between poor stress management and inadequate self-care practices in hypertensive individuals, with those having poor stress management being three times more likely to have poor self-care. This finding is consistent with previous research, including a study in Debre Tabor, which also showed a significant link between stress management and self-care practices (18). Stress impedes positive health behaviors and worsens hypertension by harming mental health, reducing motivation, leading to unhealthy behaviors, and impairing self-care and decision-making. Chronic stress can further elevate blood pressure, complicating hypertension management. Interventions for hypertensive patients should focus on stress management techniques like mindfulness-based stress reduction (MBSR), cognitive behavioral therapy (CBT), relaxation exercises, and yoga to reduce stress, improve blood pressure, and boost self-care and well-being.

The study found that individuals first diagnosed with high blood pressure during routine check-ups were 7.7 times more likely to have poor self-care practices than those diagnosed after developing complications. This is consistent with findings from Saudi Arabia (24). Being first diagnosed with high blood pressure during routine check-ups is linked to poor self-care due to limited time to grasp the condition’s long-term implications, reduced urgency, and potentially insufficient education on hypertension management. Patients with complications are likely more concerned about managing their hypertension than those newly diagnosed, as they experience the direct impact of poorly controlled hypertension. This motivates them to prioritize better management and adhere to treatment strategies. Enhancing awareness and understanding can encourage proactive self-care, resulting in better long-term health outcomes.

## Limitations of the study

The generalizability of our study is strengthened by its random sampling and inclusion of diverse health institutions settings across a broad geographic area. However, the cross-sectional design limits the ability to establish causal relationships between factors and self-care practices. Additionally, self-reported data may be subject to recall and social desirability biases, potentially affecting accuracy.

## Conclusion

This study highlights significant associations between various factors and self-care practices among individuals with hypertension. A notable proportion demonstrated inadequate self-care practices, emphasizing the necessity for targeted interventions. Factors such as education level, marital status, hypertension knowledge, stress management, and timing of diagnosis were identified as critical determinants. Moving forward, healthcare providers should prioritize tailored educational interventions, stress management strategies, and early detection efforts to enhance hypertension awareness and management. Community-based support programs offer promise in addressing psychosocial factors influencing self-care. By addressing these factors comprehensively, healthcare systems can empower individuals to adopt proactive self-care behaviors, thus improving long-term health outcomes for hypertensive populations.

## Data Availability

The raw data supporting the conclusions of this article will be made available by the authors, upon reasonable request.
